# Genome Sequence of Bivens Arm Virus, a Tibrovirus Belonging to the Species Tibrogargan virus (*Mononegavirales*: *Rhabdoviridae*)

**DOI:** 10.1128/genomeA.00089-15

**Published:** 2015-03-19

**Authors:** Michael Lauck, Shuǐqìng Yú, Yíngyún Caì, Lisa E. Hensley, Charles Y. Chiu, David H. O’Connor, Jens H. Kuhn

**Affiliations:** a University of Wisconsin-Madison, Madison, Wisconsin, USA; b Integrated Research Facility at Fort Detrick, National Institute of Allergy and Infectious Diseases, National Institutes of Health, Fort Detrick, Frederick, Maryland, USA; c University of California, San Francisco, San Francisco, California, USA

## Abstract

The new rhabdoviral genus *Tibrovirus* currently has two members, Coastal Plains virus and Tibrogargan virus. Here, we report the coding-complete genome sequence of a putative member of this genus, Bivens Arm virus. A genomic comparison reveals Bivens Arm virus to be closely related to, but distinct from, Tibrogargan virus.

## GENOME ANNOUNCEMENT

The mononegaviral family *Rhabdoviridae* currently includes 11 genera (1). The recently established genus *Tibrovirus* includes two species, Tibrogargan virus and Coastal Plains virus, for Tibrogargan virus (TIBV) and Coastal Plains virus (CPV) members, respectively (1). TIBV was isolated in 1976 from biting midges of the species Culicoides brevitarsis Kieffer (Diptera: Ceratopogonidae) in Peachester, Queensland, Australia (2). CPV was isolated in 1981 from the blood of a healthy steer at the Coastal Plains Research Station, Northern Territory, Australia (3). Antigenic comparisons established the relationship between TIBV and CPV (3). Later genomic characterization revealed them to possess three unique putative genes (designated U1, U2, and U3) and to be distinct members of the dimarhabdovirus supergroup (4). In 1989, Gibbs et al. (5) reported the isolation of Bivens Arm virus (BAV) from biting midges of the species Culicoides insignis Lutz captured in 1981 in FL, and they established an antigenic relationship between this virus, TIBV, CPV, and another putative tibrovirus, Sweetwater Branch virus (5). Serological surveys suggest that all four viruses are primarily transmitted by culicoids that dwell among bovids (2, 3, 5–7). None of the four viruses are known to cause disease in any animal, but a virus more distantly related to them, Bas-Congo virus, is speculated to infect and cause severe disease in humans (8).

To evaluate whether BAV is indeed a tibrovirus, we obtained the BAV UF 10 isolate from the World Reference Center for Emerging Viruses and Arboviruses (WRCEVA) (Galveston, TX) and grew a virus stock in Aedes albopictus C6/36 cells (U.S. Department of Agriculture, Manhattan, KS) cultured at 28°C and 5% CO_2_ in Eagle’s minimum essential medium (Lonza, Allendale, NJ) containing 2% fetal bovine serum (Sigma, St. Louis, MO). Virus RNA isolation and cDNA synthesis from cleared culture supernatants were performed as previously described (9). The Nextera XT kit (Illumina, San Diego, CA) was used to prepare deep-sequencing libraries. Sequencing was performed on a MiSeq (Illumina, San Diego, CA). CLC Genomics Workbench 7.5 (CLC bio, Aarhus, Denmark) was used to remove low-quality (Phred score, <Q30) and short reads (<100 bp). The remaining reads were mapped to the genome of Tibrogargan virus isolate CS(IRO) 132 (GenBank accession no. GQ294472.1, RefSeq no. NC_020804.1), resulting in 7,740-fold average coverage.

The determined BAV coding-complete genome sequence was found to be ≈93% identical to that of TIBV and contains all of the tibrovirus-typical open reading frames. Based on these data, we confirm that BAV is a tibrovirus that most likely should be assigned to the existing species Tibrogargan virus.

### Nucleotide sequence accession number.

The GenBank accession number of Bivens Arm virus isolate UF 10 is KP688373.
